# Analysis of the Rule of TCM Compatibility in TCM Prescriptions Containing *Ginseng Radix ET Rhizoma* in Ancient Books for *Xiaoke Bing*

**DOI:** 10.1155/2020/9472304

**Published:** 2020-03-27

**Authors:** Xiuli Sun, Bo Zhang, ShuHua Wang, Shuying Liu, Qingying Zhou

**Affiliations:** ^1^Jilin Ginseng Academy, Changchun University of Chinese Medicine, Changchun1 30117, Jilin, China; ^2^College of Computer Science and Technology, Northeast Normal University, Changchun 130117, Jilin, China; ^3^Department of Jingui, Basic Medical College, Changchun University of Chinese Medicine, Changchun 130117, Jilin, China

## Abstract

**Background:**

TCM considers that diabetes belongs to the scope of *Xiaoke Bing*. Compound prescriptions are characteristics of TCM. For a certain medicine, its compatibility with different medicines can exert different efficacies in different prescriptions. Using the TCM compound prescriptions containing *Ginseng Radix ET Rhizoma* in ancient books for *Xiaoke Bing* as an example, this study introduces new methods to investigate the rule of TCM compatibility.

**Methods:**

Frequency analysis was accomplished by programs written in Perl. The R, Cytoscape, and DpClus software were used to carry out the association rules analysis, the construction of the TCM interconnection network, and the graph clustering analysis, respectively.

**Results:**

Frequency analysis ranked the frequencies of medicine, medicinal flavors, properties, and meridian attributions, and it was found that some of them are significantly higher than others. Six association rules were obtained. The TCM interconnection network showed that there are close medicine associations among prescriptions, and we got 17 categories of closely related prescriptions from the network.

**Conclusions:**

*Ginseng Radix ET Rhizoma* was widely used in treating *Xiaoke Bing*. Our results are consistent with the understanding of *Xiaoke Bing* in TCM; hence, it is demonstrated that the methods are effective for exploring the rule of TCM usage in prescriptions. This analysis could provide references for the treatment of diabetes.

## 1. Introduction

Because of the advantages and characteristics of traditional Chinese medicine (TCM), TCM compound prescriptions have been widely used for thousands of years. There are many factors controlling the efficacies of medicines, among which the most important factor is the performance of the compatible medicine. The compatibilities of a medicine with different medicines can induce differential effects. Therefore, it is necessary to further study TCM compound prescriptions, especially for prescriptions that have been used for a long time, and to summarize the rule of TCM usage across different prescriptions. Such analysis may be helpful for enhancing the accuracy of treatments with clinical medications.

*Panax ginseng* Meyer (hereinafter referred to as *P. ginseng* as a plant and *Ginseng Radix ET Rhizoma* as a medicine in TCM) is one of the most valuable medicinal plants and is widely used in East Asia and North America, especially in Korea, China, and Japan [1]. *P. ginseng* has been used in TCM for more than 2000 years. *P. ginseng* and its components have been shown to exhibit a variety of pharmacological activities [2–4] through different mechanisms and pathways *in vitro*, *in vivo*, and in clinical models and have therapeutic effects [5–10] in a variety of human diseases. In recent years, with an increased focus on ginseng chemical components, the hypoglycemic activity of ginseng and its active ingredients has been gradually recognized by researchers. Niu et al. [11, 12] studied the effects of ginsenoside 20(S)-Rg3 and ginseng polysaccharide in type-2 diabetic rats from the perspective of metabolomics by rapid liquid chromatography-mass spectrometry. Jiao et al. [13] studied the changes of antihyperglycemic activity of ginseng pectin induced by chemical and heat treatment, and Liu et al. [14] studied the therapeutic effects of malonyl ginsenosides on type-2 diabetic rats induced by high-fat diet and streptozotocin. The inspiration of these studies originates mainly from the treatment of *Xiaoke Bing* by *P. ginseng* in ancient books.

*Xiaoke Bing* in TCM is characterized by excessive drinking, eating, polyuria, emaciation or turbidity of urine, and sweetness of urine. Thus, TCM considers that diabetes belongs to the scope of *Xiaoke Bing*. TCM has a long history of knowledge of *Xiaoke Bing*. As early as the second century B.C., *Huangdi Neijing*, the earliest TCM masterpiece in China, pioneered the understanding of *Xiaoke Bing*. With the development of later generations, a complete diagnosis and treatment system has been formed [15]. There are a large number of TCM compound prescriptions containing ginseng (while as a Chinese medicine, it is called *Ginseng Radix ET Rhizoma*) to treat *Xiaoke Bing* in ancient books. Collecting and sorting out these prescriptions and elucidating their rule of TCM usage across different prescriptions may be of clinical significance for further guiding the treatment of modern diabetes.

## 2. Materials and Methods

The prescriptions studied in this paper originate from ancient books of TCM, before the Republic of China included them in *the Chinese Medical Code* (*Fifth Edition*). First, we retrieved the *Chinese Medical Code* with 35 keywords of different kinds and names of ginseng that appeared at different periods and constructed a database for retrieving the results. Second, the database was searched with 21 keywords representing different types and symptoms of *Xiaoke Bing* that were summarized from the ancient books of TCM. Finally, a clinician filtered the prescriptions to determine the final data.

The inclusion criteria of prescriptions were as follows: a clear prescription composition, dosage, and clinical indications; the main prescriptions had to contain *Ginseng Radix ET Rhizoma*; and the original prescription for clinical addition and subtraction had to contain *Ginseng Radix ET Rhizoma*.

### 2.1. Data Preprocessing

Because different books and different historical periods have different appellations for the same medicine, the medicine names of constituent prescriptions have been unified according to *the Chinese Pharmacopoeia* and *the Great Dictionary of Traditional Chinese Medicine*. If a medicine was not included in these two books, the medicine name was unified according to provincial and ministerial local standards. TCMs processed by different methods were treated as different medicines.

The dosage of each medicine was not considered in the following analysis.

### 2.2. Frequency Analysis

In the present study, the computer programming language, Perl [16], was used to program the frequency analysis of all prescriptions, single medicines, and medicine combinations, as well as medicinal flavors, properties, and meridian tropisms.

### 2.3. Association Rules Analysis

The association rules of medicines in prescriptions were analyzed by using the *arules* package in the open-source software, R [17–19]. The resulting association rules were in the form of X ⟶ Y, in which X represents LHS items and Y represents RHS items. Each association rule involved two indicators. The support degree denoted the probability that LHS and RHS were present in one prescription. The confidence level denoted the probability of the appearance of RHS in a prescription on the basis of LHS appearing in the same prescription. These two indicators reflected the drug compatibility tendency in statistics.

### 2.4. TCM Interconnection Network Construction

The TCM interconnection network consisted of nodes and edges that represented the following: nodes represented medicines, the size of which represented the frequency of medicine occurrence in all prescriptions; the edge connecting two medicines represented that both medicines appeared in one prescription; and the width of the edge represented the frequency of this appearance. The final TCM interconnection network was displayed by Cytoscape software [20].

### 2.5. Graph Clustering Analysis

To identify the densely connected node groups in the TCM interconnection network (i.e., to identify highly overlapping prescription groups and to analyze the subtle differences of prescription medication), we used DPClus [21] software to perform graph clustering analysis. DPClus tends to isolate densely connected regions of a graph as clusters.

## 3. Results and Discussion

### 3.1. Prescriptions Overview

In total, 303 prescriptions were ultimately obtained, and the authors and works of more than 10 corresponding prescriptions are shown in Table 1.

The number of repetitions of prescriptions may partly explain their wide applications. The most repeated prescription consisted of *Rubi Fructus*, *Coptidis Rhizoma*, *Galli Gigerii Endothelium Corneum*, *Ginseng Radix ET Rhizoma*, *Cnidii Fructus*, *Dendrobii Caulis*, *Rehmanniae Radix Praeparata*, *Trichosanthis Radix*, *Scrophulariae Radix*, *Poria,* and *Dioscoreae Spongiosae Rhizoma*. This prescription has been named *Bai Fuling Pill* and has been mainly used to treat *Xiaoke Bing* of the kidney type, which is manifested as the legs gradually thinning and the waist and feet becoming weak. The second-most repeated prescription was the prescription composed of *Ginseng Radix ET Rhizoma* and *Trichosanthis Radix*, and its main use had been for treating thirst and excessive drinking.

**Figure 1:**
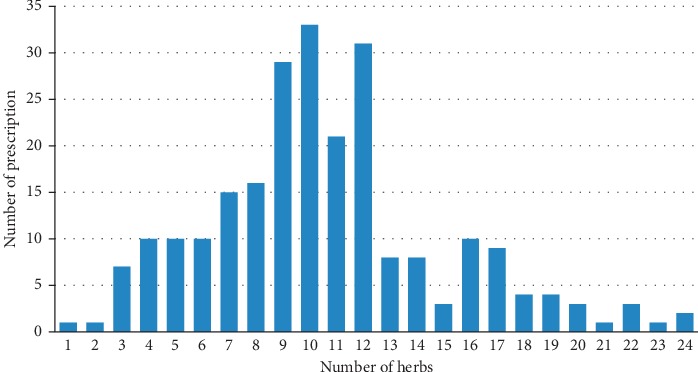
The distribution of the number of medicines in prescriptions.

The distribution of the number of medicines in prescriptions is shown in Figure 1. The average number of medicines per prescription was 10. Most prescriptions contained 9–12 medicines.

### 3.2. Frequency Analysis

There were a total of 244 medicines involved in all of the prescriptions. Aside from *Ginseng Radix ET Rhizoma* appearing in all prescriptions, the top-five most frequently used medicines were in the following order: *Ophiopogonis Radix*, *Glycyrrhizae Radix ET Rhizoma*, *Poria*, *Trichosanthis Radix*, and *Anemarrhenae Rhizoma*. Medicines with frequencies exceeding 25% are shown in Table 2.

**Figure 2:**
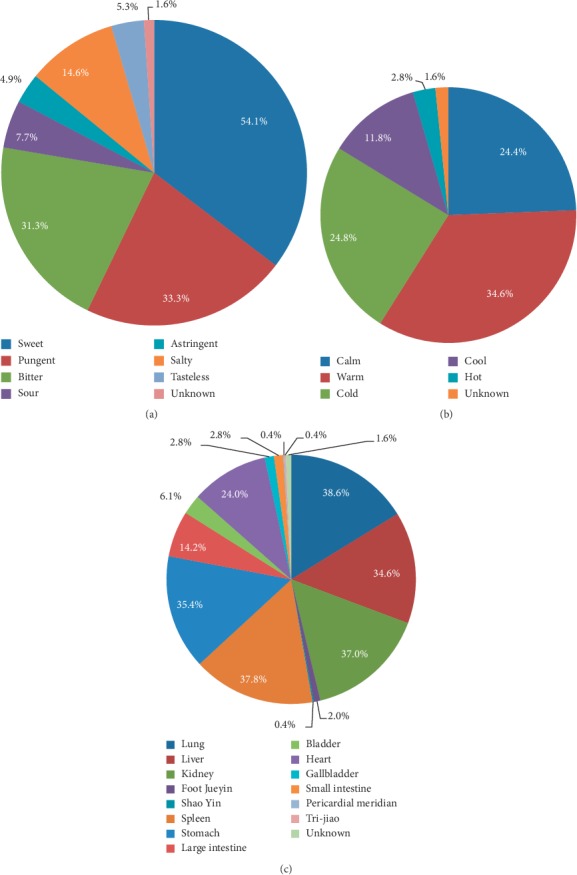
The frequency distribution of TCM properties, flavors, and meridian tropisms. (a) TCM flavors. (b) TCM properties. (c) TCM meridian tropisms.

The frequency distribution of TCM flavors is shown in Figure 2(a), in which sweet, pungent, and bitter were the dominant flavors. The frequency distribution of TCM properties is shown in Figure 2(b), in which warm, cold, and calm were the dominant properties. The frequency distribution of herbal meridian tropisms is shown in Figure 2(c), in which the lungs, spleen, kidney, stomach, and liver meridians were the most dominant. There are 4 (1.6%) herbs not included in *the Chinese Pharmacopoeia* and *the Great Dictionary of Traditional Chinese Medicine*, so their flavors, properties, and meridian tropisms are not counted, and we label them as group “unknown” in Figure 2.

The following four groups of three-medicine combinations each had a frequency of more than 20%: *Glycyrrhizae Radix Et Rhizoma*, *Ophiopogonis Radix*, and *Ginseng Radix Et Rhizoma* (0.25%); *Poria*, *Ophiopogonis Radix*, and *Ginseng Radix ET Rhizoma* (0.25%); *Astragali Radix*, *Ophiopogonis Radix*, and *Ginseng Radix ET Rhizoma* (20%); and *Poria*, *Glycyrrhizae Radix ET Rhizoma*, and *Ginseng Radix ET Rhizoma* (20%). The following three groups of four-medicine combinations each had a frequency of more than 10%: *Poria*, *Ophiopogonis Radix*, *Schisandrae Chinensis Fructus*, and *Ginseng Radix ET Rhizoma* (12%); *Glycyrrhizae Radix Et Rhizoma*, *Gypsum Fibrosum*, *Anemarrhenae Rhizoma*, and *Ginseng Radix ET Rhizoma* (12%); and *Glycyrrhizae Radix Et Rhizoma*, *Puerariae Lobatae Radix*, *Trichosanthis Radix*, and *Ginseng Radix ET Rhizoma* (11%).

### 3.3. Association Rules Analysis

Six association rules were obtained after setting the support degree to be greater than 0.1 and the confidence level to be greater than 0.6, as shown in Table 3. Taking rule *Poria* and *Puerariae Lobatae Radix* ⟶ *Glycyrrhizae Radix Et Rhizoma* as an example, its support degree was 0.1, which denotes that the probability of three medicines appearing simultaneously in a TCM compound prescription was 10% and that its confidence degree was 1. This indicates that when *Poria* and *Puerariae Lobatae Radix* are included in a TCM compound prescription, there is a 100% possibility of *Glycyrrhizae Radix ET Rhizoma* also appearing in the same prescription. Moreover, association rule analysis may help to identify the commonly used medicine compatibilities. And from the 6 rules, we can find that the *Glycyrrhizae Radix Et Rhizoma*, *Puerariae Lobatae Radix*, *and Poria* are the most commonly used medicine compatibilities.

### 3.4. Construction of TCM Interconnection Network

**Figure 3:**
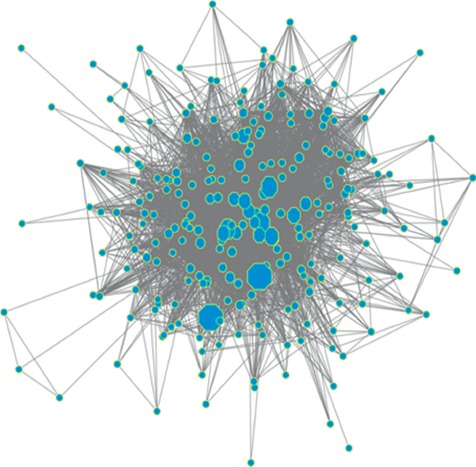
TCM interconnection network. The size of the node represents the frequency of the medicine.

Since *Ginseng Radix ET Rhizoma* appeared in every prescription, all of its associations were removed in the construction of the TCM interconnection network. The final network consisted of 243 nodes and 3,552 edges, as shown in Figure 3.

As can be seen from Figure 3, the nodes in the middle part of the network were closely related and were loosely surrounded. This pattern indicates that there are some prescriptions that mostly contain the same medicines. Identifying these prescription groups and elucidating their rules and subtle differences may help in providing more accurate application of TCM.

### 3.5. Graph Clustering Analysis

Using the parameters *d*_min_ = 0.9, cp_min_ = 0.7, and minimum cluster size = 10, the graph cluster analysis extracted 24 clusters from the TCM interconnection network. Except for 7 ineffective clusters corresponding to only one TCM compound prescription, the other 17 clusters of the prescription were highly coincident in the inclusion of medicines.

**Figure 4:**
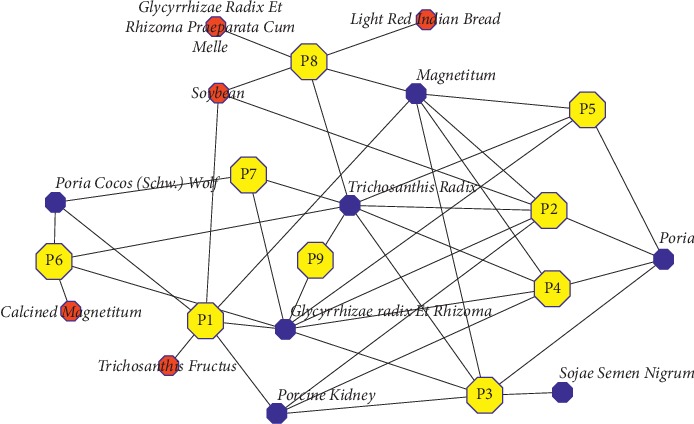
Interrelation of the nine prescriptions corresponding to cluster 21. Yellow nodes represent prescriptions, blue nodes represent medicines gathered in cluster 21, and red nodes represent medicines not in cluster 21.

For example, cluster 21 had 12 nodes, including *Adenophora Trachelioides Maxim*, *Sojae Semen Nigrum*, *Magnetitum*, *Glycyrrhizae Radix Et Rhizoma*, *Trichosanthis Radix*, *Scutellariae Radix*, *Poria*, *Anemarrhenae Rhizoma*, *Puerariae Lobatae Radix*, *Poria Cocos* (*Schw*.) *Wolf*, *Gypsum Fibrosum*, and *Porcine Kidney*, which corresponded to nine prescriptions. The *Zhushenqini solution* in *Qixiao Liangfang* (number P1) and the *Zhushenqini solution* in *Zhengzhi Zhunsheng-Leifang* (number P2) are used to treat *Xiaozhong*. The *Zhushenqini solution* in *Compendium of Materia Medica* (number P3), the nameless prescription to treat *Xiaoke Bing* in *Zabing Yuanliu Xizhu* (number P4), the *Shiziqini solution* in *Zabing Yuanliu Xizhu* (number P5), and the *Shiziqini solution* in *Puji Fang* (number P6) are used for *Xiaoke Bing* of the *Qiangzhong* type. The *Qini powder* in *Taiping Shenghui Fang* (number P7), *magnetite pill* in *Shengji Zonglu* (number P8), and *Qini powder* in *Puji Fang* (number P9) are used for *Xiaozhong* heat vexation. These nine prescriptions all contained *Ginseng Radix ET Rhizoma*, *Puerariae Lobatae Radix*, *Gypsum Fibrosum*, *Scutellariae Radix*, *Anemarrhenae Rhizoma*, and *Adenophora Trachelioides Maxim*. In addition to these common medicines, the subtle differences of medicines among these prescriptions are shown in Figure 4.

In summary, the related prescriptions of a cluster had the following characteristics. First, they contained most of the same medicines, which represented the same main efficacies of the prescriptions. The small number of different medicines suggests that the symptoms treated by these prescriptions belong to a specific type of disease or due to the similar etiology and/or pathogenesis, or the doctors at each time period may have adjusted and added or subtracted the prescriptions for treating different clinical manifestations of the same disease syndrome, or simply because doctors have different medication habits for the same disease. This analysis is of great significance for studying the development and change of the same disease over different periods and the rule of medicinal usage for the same disease in different patients or different stages of development. Second, the prescriptions appearing in different ancient books with the same name may represent the same prescription in the process of inheritance in different periods. This is of great significance to the study of the historical evolution and traceability of a prescription.

## 4. Discussion

For the high-frequency medicines that we identified, their efficacies for treating primary symptoms were in accordance with the symptoms of *Shangxiao* and *Xiaxiao* of TCM; their meridian tropisms conformed to the understanding of the location and classification of *Xiaoke Bing* in TCM; their flavors and properties were in line with the understanding of the etiology and pathogenesis of *Xiaoke Bing* in TCM.

The disease location of *Xiaoke Bing* is closely related to the lung, spleen, stomach, and kidney, and according to these locations, TCM has divided *Xiaoke Bing* into the following three categories: *Shangxiao*, *Zhongxiao*, and *Xiaxiao*. The etiology and pathogenesis consist of impairment of body fluid due to lung heat, excessiveness of stomach fire, and uncontrolling of the losses or overflow of *Qi*, blood, and body fluid in the body due to kidney deficiency. According to our results, *Gypsum Fibrosum*, *Anemarrhenae Rhizoma*, *Trichosanthis Radix*, *Scrophulariae Radix*, and *Coptidis Rhizoma* may ameliorate the fire of the lung and stomach and help to produce saliva and to slake thirst. *Ophiopogonis Radix*, *Dendrobii Caulis*, and *Rehmanniae Radix Praeparata* can nourish *Yin*, supply essence, clear heat, and moisten dryness. *Glycyrrhizae Radix ET Rhizoma* and *Poria* can tonify the spleen. *Schisandrae Chinensis Fructus*, *Rubi Fructus*, *Galli Gigerii Endothelium Corneum*, and *Cnidii Fructus* have the effect of astringent therapy, convergence, and can control semen. *Ginseng Radix ET Rhizoma* is compatible with the above TCMs to produce both *Qi* and *Yin* and to supply both *Qi* and body fluid to achieve the effect of balancing *Yin* and *Yang*. Exploring the rule of TCM usage in the treatment of *Xiaoke Bing* in ancient books of TCM, combined with the different clinical manifestations of modern diabetes patients, may provide further reference for the medicinal treatment of modern diabetes.

## 5. Conclusions

Our results showed that *P. ginseng* has been widely used in treating *Xiaoke Bing*, and there were different medicine combinations for different types of syndromes. The research analysis and results demonstrate that the methods and software introduced in this present study may effectively analyze the rule of usage of TCM medications within prescriptions.

## Figures and Tables

**Table 1:** Authors, works, and the number of corresponding prescriptions.

Works	Authors	Number of prescriptions
Puji Fang	Di Zhu	50
Shengji Zonglu	Ji Zhao	28
Zabing Yuanliu Xizhu	Jin‘ao Shen	23
Taiping Shenghui Fang	Huaiyin Wang, You Wang, Zhaoyu Chen, Qi Zheng et al.	21
Qixiao Liangfang	Su Dong, Xian Fang	16
Jifeng Puji Fang	Rui Zhang	14
Gujin Yitong Daquan	Chunfu Xu	11

**Table 2:** Medicines with frequencies exceeding 25%.

Medicine	Frequency
*Ophiopogonis Radix*	121 (50%)
*Glycyrrhizae Radix Et Rhizoma*	110 (45%)
*Poria*	100 (41%)
*Trichosanthis Radix*	83 (34%)
*Anemarrhenae Rhizoma*	69 (28%)
*Astragali Radix*	67 (27%)
*Schisandrae Chinensis Fructus*	66 (27%)
*Puerariae Lobatae Radix*	62 (25%)
*Scutellariae Radix*	61 (25%)
*Coptidis Rhizoma*	60 (25%)

**Table 3:** Table of association rules.

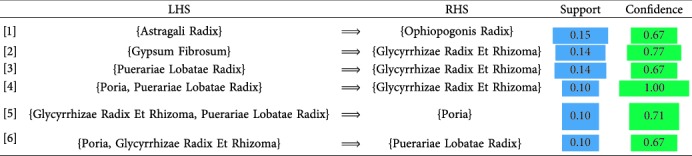

## Data Availability

The data used to support the findings of this study are available from the corresponding author upon request.
